# Involvement of NO in V-ATPase Regulation in Cucumber Roots under Control and Cadmium Stress Conditions

**DOI:** 10.3390/plants12152884

**Published:** 2023-08-07

**Authors:** Magdalena Zboińska, Anna Janeczko, Katarzyna Kabała

**Affiliations:** 1 Department of Plant Molecular Physiology, Faculty of Biological Sciences, University of Wrocław, Kanonia 6/8, 50-328 Wrocław, Poland; magdalena.zboinska@uwr.edu.pl; 2 The Franciszek Górski Institute of Plant Physiology, Polish Academy of Sciences, Niezapominajek 21, 30-239 Krakow, Poland; a.janeczko@ifr-pan.edu.pl

**Keywords:** cadmium, hydrogen peroxide, hydrogen sulfide, nitric oxide, nitrate reductase, salicylic acid, V-ATPase

## Abstract

Nitric oxide (NO) is a signaling molecule that participates in plant adaptation to adverse environmental factors. This study aimed to clarify the role of NO in the regulation of vacuolar H^+^-ATPase (V-ATPase) in the roots of cucumber seedlings grown under control and Cd stress conditions. In addition, the relationship between NO and salicylic acid (SA), as well as their interrelations with hydrogen sulfide (H_2_S) and hydrogen peroxide (H_2_O_2_), have been verified. The effect of NO on V-ATPase was studied by analyzing two enzyme activities, the expression level of selected *VHA* genes and the protein level of selected VHA subunits in plants treated with a NO donor (sodium nitroprusside, SNP) and NO biosynthesis inhibitors (tungstate, WO_4_^2^**^−^** and N-nitro-L-arginine methyl ester, L-NAME). Our results indicate that NO functions as a positive regulator of V-ATPase and that this regulation depends on NO generated by nitrate reductase and NOS-like activity. It was found that the mechanism of NO action is not related to changes in the gene expression or protein level of the V-ATPase subunits. The results suggest that in cucumber roots, NO signaling interacts with the SA pathway and, to a lesser extent, with two other known V-ATPase regulators, H_2_O_2_ and H_2_S.

## 1. Introduction

It is estimated that approximately 30,000 t of cadmium (Cd) are released into the environment each year, mostly as a result of anthropogenic activity. This large amount of Cd accumulates primarily in the surface layers of the soil, where it is absorbed by the roots, inducing a stress response in plant organisms [1]. The easily noticeable effects of Cd toxicity include stunted growth, thickening of roots, chlorosis, leaf curling, reduction of leaf blade area and acceleration of senescence [2,3,4]. At the cellular level, cadmium generates oxidative stress and directly binds to cellular components, damaging the structure of DNA, proteins, and lipids, consequently leading to disorders in biological membranes [2].

In response to a stress factor, plants activate defense mechanisms that allow them to adapt to unfavorable conditions and function despite the penetration of harmful factors into the cells [2]. However, under long-term exposure to Cd, these mechanisms are not sufficient to protect cells from the negative effects of the metal. Among others, Cd was found to inhibit vacuolar H^+^-ATPase (V-ATPase) [5,6,7,8,9,10,11,12], one of the proton pumps of plant cells. Using ATP as an energy source, this enzyme transfers protons across the tonoplast, as well as the membranes of the Golgi apparatus and early endosomal networks [13,14]. ATPase-dependent H^+^ accumulation decreases the pH of the organelle lumen, which is necessary for efficient functioning of vacuolar hydrolytic enzymes or secretory cargo transport. In addition, it ensures solute homeostasis in the cell because the proton electrochemical gradient is a driving force for many symporters and antiporters [15]. 

Disturbance of pump activity, which results in inhibition of cell growth [16], probably also reduces the detoxification capacity of the cytosol by inhibiting the translocation of metal ions into the vacuole. In addition to primary active transporters, the accumulation of Cd^2+^ in this compartment is supported by secondary active transporters, including CAX2 and CAX4 exchangers described in *Arabidopsis thaliana* [17] and metal tolerance proteins MTP1 and MTP4 identified in tobacco [18]. Therefore, maintaining the activity of the proton pump under cadmium stress is extremely important. It was shown that heterologous overexpression of only one V-ATPase gene, *VHA-c1*, from *Tamarix hispida* significantly increased *Arabidopsis* tolerance to Cd [19]. 

Many reports suggest that nitric oxide (NO) participates in Cd-induced stress signaling and may be involved in tolerance acquiring [20,21,22,23]. However, little is known about V-ATPase modulation by NO [9,10,11]. The signaling properties of NO come from its relatively long life, small size, and the ability to easily penetrate biological membranes, as well as the ability to easily diffuse in the hydrophilic environment of the cytoplasm. NO is also characterized by its high chemical reactivity related to its free radical nature (NO^∙^). Thus, NO reacts with many molecules, including hydroxyl radical (OH^∙^) and superoxide anion radical (O_2_**^−^**^∙^) or alkoxy (LO^∙^) and peroxyl (LOO^∙^) radicals of lipids, minimizing oxidative stress and limiting lipid peroxidation. These features determine the antioxidant properties of NO. Another target of NO is proteins [24,25]. The post-translational modifications induced by NO include Tyr nitration, Cys S-nitrosylation, and metal nitrosylation. In this way, NO regulates the activity of transcription factors, kinases, receptors, transporters, antioxidant enzymes, and enzymes responsible for phytohormone synthesis [26]. 

Regulation of NO levels in plant cells is much more complex than that in animals, and depends on many metabolic processes that are not fully understood [27]. Nitrate reductase (NR) was originally proposed to be the main enzyme involved in NO synthesis in plants because it is able to convert NO_2_**^−^** to NO using NADH as an electron source. However, many authors argue that cytoplasmic NR is responsible for NO production for signaling purposes [27,28]. An alternative hypothesis assumes that NR delivers electrons to NO-forming nitrite reductase (NOFNiR), which catalyzes NO synthesis from NO_2_**^−^**; however, this process was confirmed only in *Chlamydomonas*, but not in higher plants [29,30]. Moreover, a second form of NR, plasma membrane-bound nitrate reductase (PM-NR), probably participates in NO synthesis by providing the substrate, NO_2_, to nitrite-nitric oxide reductase (Ni-NOR), which generates NO in apoplast [31]. 

Another pathway for NO generation in plant cells appears to be Arg-dependent synthesis. In animals, this process is catalyzed by nitric oxide synthase (NOS). In plants, NOS-like activity is measured using cofactors characteristic for animal NOS and is inhibited in the presence of anti-mouse NOS antibodies [32] or Arg analogs (e.g., L-NAME) [33]. On the other hand, however, NOS-like genes have not yet been identified in land plants [34]. In addition, NO can be generated in non-enzymatic reaction between L-Arg and H_2_O_2_ [32] or in the polyamine-based pathway [33]. 

In our previous study [7], the effects of small signaling molecules, including H_2_O_2_ and H_2_S, on V-ATPase were analyzed in both control and Cd-treated cucumber roots. The relationship between these signals has been found to be involved in this enzyme regulation. The aim of this work was to clarify the role of NO in the regulation of V-ATPase and to demonstrate the connections between this signaling molecule and other potential regulators of the enzyme, including phytohormones. For this purpose, V-ATPase activity was determined in plants treated with a NO donor (SNP) or inhibitors of NO-generating enzymes. We showed that NO acts as a positive regulator of the proton pump in the roots of cucumber seedlings growing under control conditions and cadmium stress. However, Cd was found to decrease NO level in the roots. To explain the mode of NO action, the gene expression and protein level of the selected VHA subunits were determined. Based on the phytohormone content analysis in Cd-treated cucumber roots, salicylic acid (SA) was selected as a possible V-ATPase modulator. In addition, the interrelations between NO and salicylic acid as well as between NO and H_2_O_2_/H_2_S were verified to explain the possible crosstalk responsible for the observed Cd-induced negative regulation of V-ATPase.

## 2. Results

**Figure 1:**
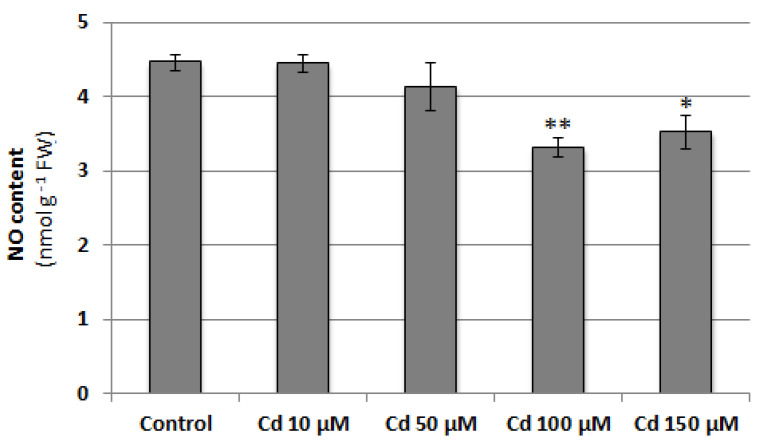
Effect of cadmium on NO content in cucumber roots. Plants were treated with 10, 50, 100, or 150 µM CdCl_2_ or grown without this metal (control) for 24 h. Data represent the means of three biological repetitions ± SE. Statistically significant differences (independent-sample *t*-test) between the control and Cd treatments are marked as * (0.01 ≤ *p* < 0.05) or ** (*p* < 0.01).

**Figure 2:**
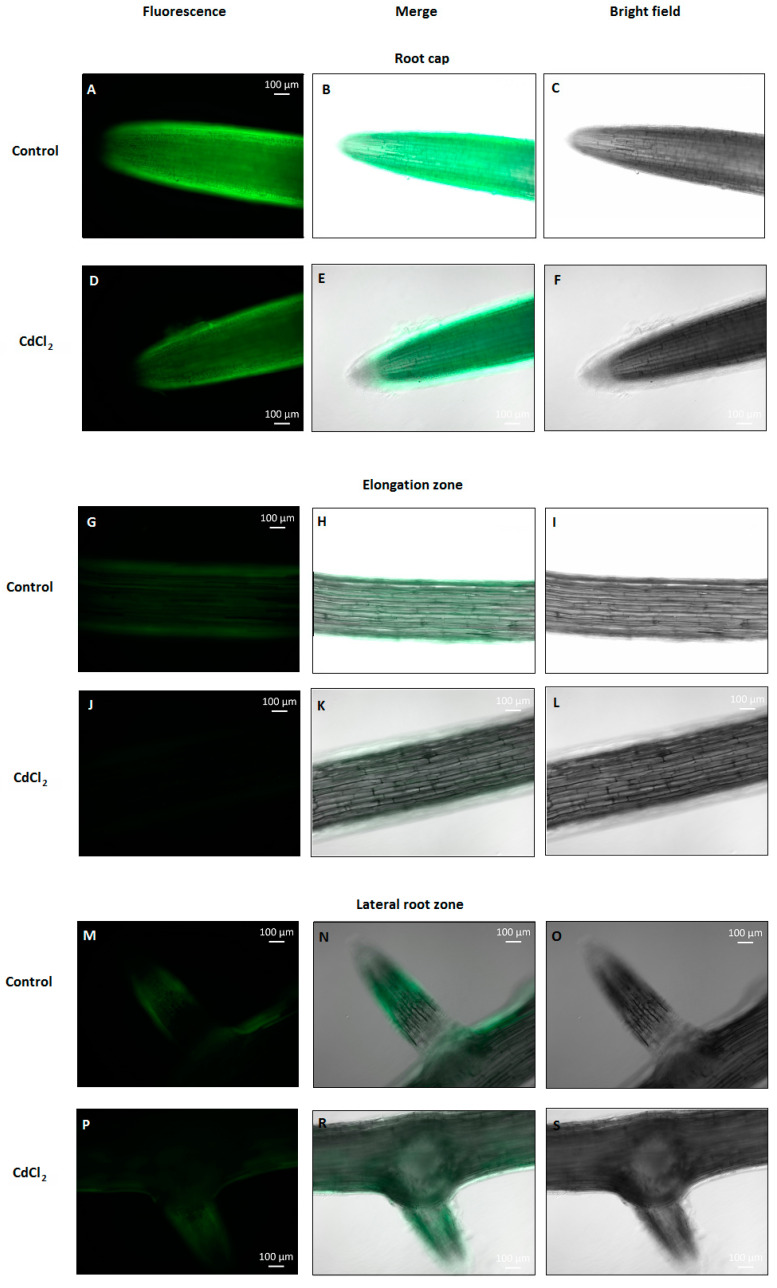
Bio-imaging of NO production in cucumber roots treated with 100 µM Cd for 24 h. The NO level was monitored using DAF-2D, a NO-specific fluorescent dye, and imaged using fluorescence microscopy. The images are representative of several measurements made in the root cap (**A**–**F**), elongation zone (**G**–**L**), and lateral root zone (**M**–**P**,**R**,**S**).

To verify whether NO content changes in cucumber roots under cadmium stress conditions, seedlings were treated with 10, 50, 100 or 150 μM CdCl_2_ for 24 h. At higher concentrations, Cd was found to significantly decrease NO level. It reached about 74% and 79% of the control value in plants exposed to 100 and 150 μM Cd, respectively (Figure 1). The negative effect of 100 μM CdCl_2_ on NO level in cucumber roots was also confirmed by DAF-2D bio-imaging (Figure 2). According to this, a Cd concentration of 100 μM was used in subsequent studies.

**Figure 3:**
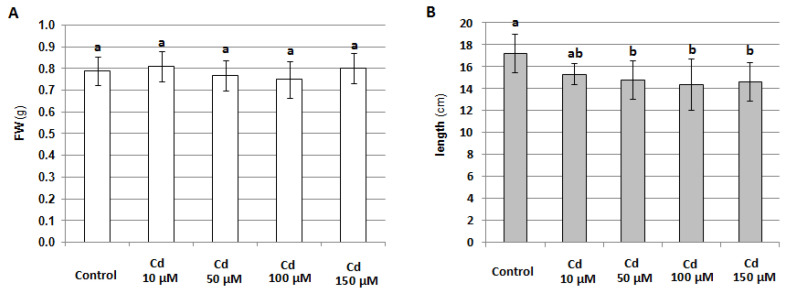
Effect of 24-hour treatment of cucumber seedlings with different concentrations of CdCl_2_ on seedling fresh weight, FW (**A**) and length (**B**). The results presented are the average of the measurements of 10 plants ± SE. Different letters represent homogeneous groups according to Tukey’s test (*p* < 0.05).

At the same time, it was shown that 24-h treatment of seedlings with 100 μM cadmium did not significantly affect their phenotype (Figure S1) or fresh weight (Figure 3A); however, higher Cd concentration reduced seedling elongation (Figure 3B). On the other hand, prolonged exposure toe 100 μM metal led to a marked impairment in cucumber growth and development, and it seemed to reduce the lifespan (Figure S2). 

**Figure 4:**
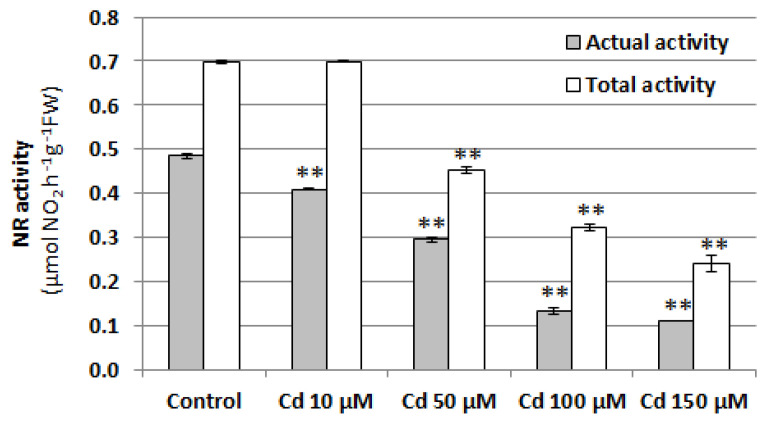
Effect of cadmium on NR activity in cucumber roots. Plants were treated with 10, 50, 100, or 150 µM CdCl_2_ or were grown without this metal (control) for 24 h. Data represent the means of three biological repetitions ± SE. Statistically significant differences (independent-sample *t*-test) between the control and Cd treatments are marked as ** (*p* < 0.01).

Since nitrate reductase (NR) is believed to be the main enzyme responsible for NO production in plant cells, its activity was determined in the roots of cucumber seedlings treated with 10, 50, 100, or 150 μM CdCl_2_ for 24 h. Similarly to the NO level, both NR activities, total and actual, were reduced by Cd in a concentration-dependent manner. Exposure to 100 µM Cd resulted in a more than three-fold decrease in actual NR activity and an almost two-fold decrease in total NR activity (Figure 4). This suggests that the reduction in the NO level under Cd stress may be due to NR downregulation.

Additionally, to determine the dynamics of changes in Cd-induced endogenous NO, its level was monitored for 24 h after plant transfer to a medium containing 100 μM Cd. The results showed a decrease in the NO level after the first 2 h of stress factor application. A similar effect was observed in roots throughout the 24-h stress response (Figure S3).

**Figure 5:**
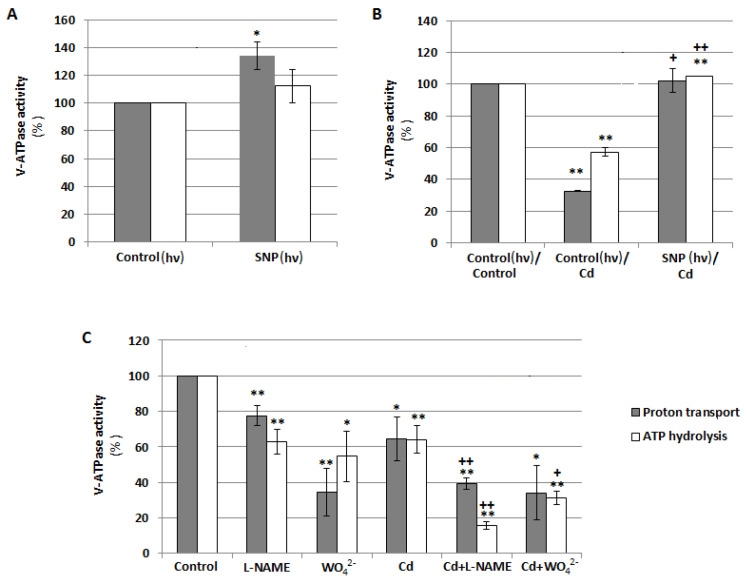
Effects of NO on V-ATPase activities, ATP-dependent proton transport and ATP hydrolysis, in tonoplast vesicles isolated from cucumber roots. (**A**) Plants were grown with the addition of 10 μM sodium nitroprusside (SNP, NO donor) or without this compound (control) for 24 h under continuous light (hν). (**B**) Plants were treated with 100 μM CdCl_2_ for 24 h (Cd) or 10 μM SNP for 24 h before exposure to 100 μM CdCl_2_ (SNP/Cd, pretreatment). Control seedlings were grown in a basic medium. During the 24-h treatment of some seedlings with SNP, all plants were transferred to continuous light conditions (hν). (**C**) Plants were exposed to 100 μM CdCl_2_, 50 μM tungstate (WO_4_^2^**^−^**, nitrate reductase inhibitor), or 100 µM N-nitro-L-arginine methyl ester (L-NAME, an inhibitor of NOS-like activity), as well as to CdCl_2_ together with WO_4_^2^**^−^** or L-NAME (Cd+WO_4_^2^**^−^** and Cd+L-NAME, respectively) for 24 h. Data represent the means of 3–6 biological repetitions ± SE. Statistically significant differences (single-sample *t*-test) related to the control are indicated as * (0.01 ≤ *p* < 0.05) or ** (*p* < 0.01) and related to Cd as + (0.01 ≤ *p* < 0.05) or ++ (*p* < 0.01).

To explain the role of NO in the regulation of V-ATPase, the effects of exogenously applied SNP (NO donor) and inhibitors of NO-generating enzymes (WO_4_^2^**^−^** as a NR inhibitor and L-NAME as an inhibitor of NOS-like activity) on both ATP hydrolysis and ATP-driven H^+^-transport were analyzed in tonoplast vesicles isolated from cucumber roots. Under normal growth conditions, the treatment of seedlings with SNP caused visible stimulation of ATP-driven proton transport which reached about 134% of the control (Figure 5A). Cadmium significantly reduced both enzyme activities. H^+^ translocation was inhibited by 67% whereas ATP hydrolysis by 43%, respectively. However, pretreatment of seedlings with SNP before their exposure to cadmium resulted in the restoration of the Cd-lowered activities to control values (Figure 5B). On the other hand, opposite results were observed when plants were exposed to L-NAME and WO_4_^2^**^−^**. Both inhibitors decreased ATP hydrolysis (by 37% and 45%, respectively) and proton pumping (by 22% and 66%, respectively) catalyzed by V-ATPase in unstressed seedlings. The addition of L-NAME or WO_4_^2^**^−^** to the nutrient medium together with cadmium reduced enzyme activities to a greater extent than the addition of metal alone. ATP-dependent H^+^ transport achieved about 39% and 34%, while hydrolytic activity was 16% and 31% of the control in roots treated with Cd+L-NAME and Cd+WO_4_^2^**^−^**, respectively (Figure 5C). The results indicate that NO acts as a positive regulator of V-ATPase under both normal and stress conditions, and diminishing the endogenous level of this molecule inhibits enzyme activity.

**Figure 6:**
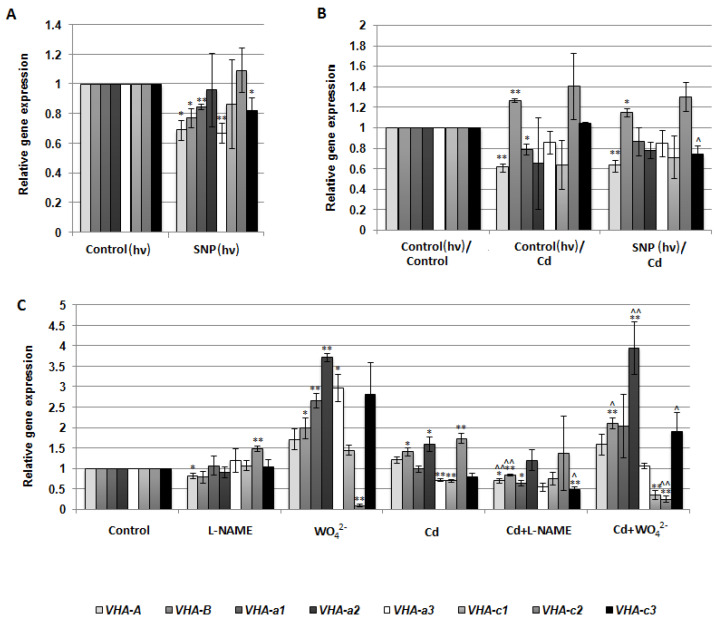
Expression level of the selected VHA genes in cucumber roots. Plants were treated with SNP, Cd, or inhibitors as described in Figure 5. (**A**–**C**) qPCR analysis was performed using two reference genes, TIP41 and EF1. Transcription level of the control was normalized to 1. Data represent the means of three biological replicates ± SE. Statistically significant differences (single-sample *t*-test) between the control and treatments are marked as * (0.01 ≤ *p* < 0.05) or ** (*p* < 0.01) and between Cd and corresponding treatments as ^ (0.01 ≤ *p* < 0.05) or ^^ (*p* < 0.01).

**Figure 7:**
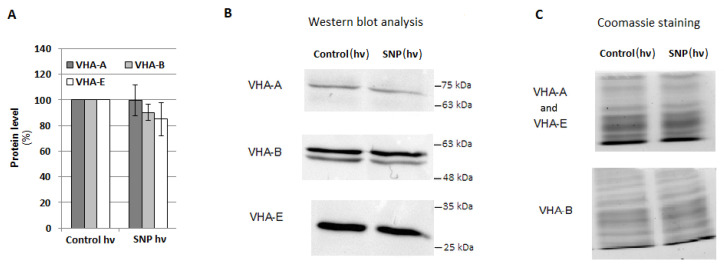
Protein level of the selected VHA subunits. Plants were treated with SNP as described in Figure 5A. (**A**) Average level of VHA-A, VHA-B, and VHA-E was analyzed by Western blotting and ImageLab™. The results are presented as a % of the protein level in the roots of the control plants (100%). Data represent the means of 5 biological repetitions ± SE. (**B**) Representative Western blot of the total protein extract with anti-VHA-A, anti-VHA-B, and anti-VHA-E antibodies. (**C**) Coomassie staining used as a protein loading control.

V-ATPase activity may be regulated at both the gene and protein level. For this reason, the expression of eight VHA genes, including *VHA-A*, *VHA-B*, *VHA-a1*, *VHA-a2*, *VHA-a3*, *VHA-c1*, *VHA-c2*, and *VHA-c3*, encoding four cucumber V-ATPase subunits, was analyzed in the roots of cucumber seedlings treated with SNP, L-NAME, WO_4_^2^**^−^**, and Cd. There were no significant correlations between proton pump activity and the transcription level of the analyzed genes, suggesting that the observed modulations of enzyme activity were not the result of changes at the genetic level (Figure 6A–C). However, the expression of some genes, especially VHA-a2, was clearly upregulated after plant exposure to WO_4_^2^**^−^**, both under control and stress conditions, although V-ATPase activity was inhibited. It seems possible that such transcriptional changes in individual subunits may be related to V-ATPase functions other than proton pumping. Moreover, treatment of cucumber seedlings with SNP did not induce any significant modulation in the protein level of the three main enzyme subunits, including A, B, and E of the cytoplasmic V_1_ sector (Figure 7A–C), suggesting that NO can modulate the proton pump at the post-translational level or activate it indirectly, and other signaling molecules may be involved in this process. 

**Figure 8:**
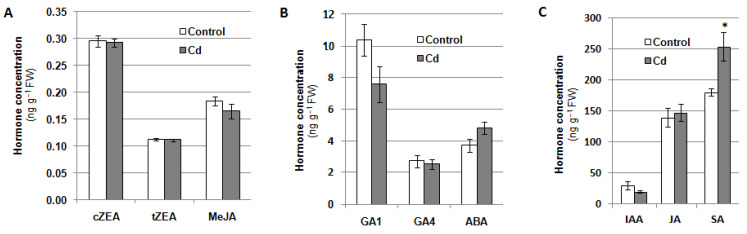
Contents of *cis*-zeatin, cZEA, *trans*-zeatin, tZEA, and methyl jasmonate, MeJA (**A**), gibberellins GA1 and GA4, and abscisic acid, ABA (**B**), indole-3-acetic acid, IAA, jasmonic acid, JA, and salicylic acid, SA, (**C**) in the roots of cucumber seedlings grown with the addition of 100 μM CdCl_2_ or without this metal (control) for 24 h. The results presented are the averages of five replicates ± SE. Statistically significant differences (independent-sample *t*-test) between the control and treatments are marked as * (0.01 ≤ *p* < 0.05).

Phytohormones are a group of signaling molecules that participate in plant responses to stress factors. Therefore, the content of the selected compounds from this group was analyzed in cucumber seedlings exposed to 100 μM cadmium for 24 h (Figure 8). It was indicated that treatment of plants with Cd visibly increased the salicylic acid (SA) level in the roots (by about 40% over the control). The content of other analysed photohormones, including auxin (IAA), cytokinins (*cis*-zeatin, cZEA, and *trans*-zeatin, tZEA), gibberellins (GA1 and GA4), abscisic acid (ABA), methyl jasmonate (MeJA), and jasmonic acid (JA), did not change significantly. At the same time, it was demonstrated that under cadmium stress, the content of benzoic acid (BeA), a precursor of SA, increased in cucumber roots (Table 1). During the 24-h exposure of the seedlings to Cd, the SA level showed two phases of increase, between 2 and 4 h as well as between 8 and 24 h after stress induction. The enhanced BeA content was partially correlated with the increase in SA concentration (Figure S4). 

**Figure 9:**
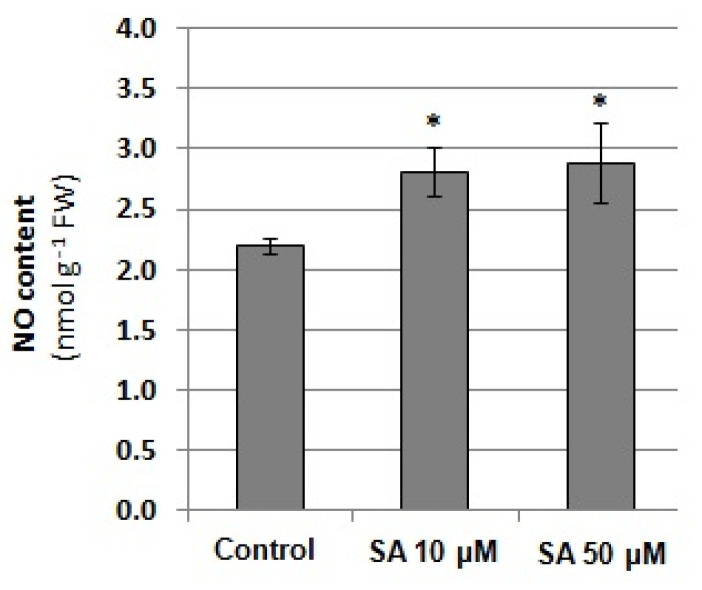
NO content in the roots of cucumber seedlings exposed to exogenous salicylic acid (SA). Plants were treated with 10 or 50 μM SA or grown without this hormone (control) for 24 h. Statistically significant differences (independent-sample *t*-test) between the control and treatments are marked as * (*p* < 0.05).

Based on the obtained results, in the next step, the relationship between NO level and SA content was verified. Exposure of seedlings to 10 and 50 µM SA for 24 h was found to enhance the NO level in cucumber roots (Figure 9). SA stimulated endogenous NO production to a similar extent regardless of the concentration used. It reached approximately 30% of the control value, suggesting that SA could act as a regulator of NO generation.

On the other hand, treatment of plants with SNP for 24 h did not affect either the SA or BeA concentrations in cucumber roots. However, the effect of NO synthesis inhibitors on SA and BeA content was observed in the roots of seedlings exposed to Cd (Table 1). The 24-h treatment of plants with WO_4_^2^**^−^**, a suppressor of NR, together with Cd caused a significant decrease in SA concentration. It achieved 73% in comparison to the level determined in roots treated with Cd alone, indicating that under cadmium stress, SA level depends on endogenous NO generated by NR. On the other hand, BeA content increased by approximately 15% in the roots of seedlings treated with L-NAME in comparison to the plants exposed to Cd. This suggests that the level of the SA precursor can also be modulated by NO, but in a different way than SA. 

**Figure 10:**
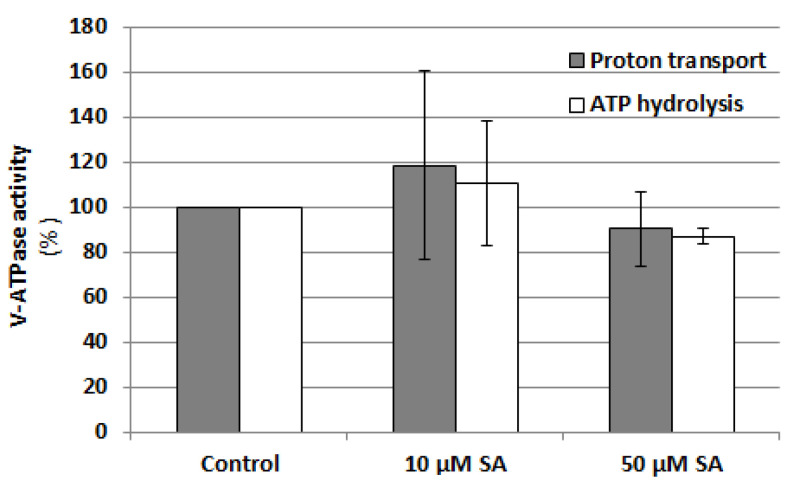
Effect of SA on V-ATPase activities, ATP-dependent proton transport and ATP hydrolysis, in tonoplast vesicles isolated from cucumber roots. Plants were grown with the addition of 10 or 50 μM salicylic acid or without this hormone (control) for 24 h. The results presented are the averages of three replicates ± SE. There were no statistically significant differences between the control and treatments (single-sample *t*-test, *p* < 0.05).

Since SA content increases under cadmium stress and both SA and NO level depend on each other, the effect of SA on V-ATPase activity was also analyzed. Cucumber seedlings were exposed to two previously used SA concentrations, 10 and 50 µM, and both ATP hydrolysis and ATP-driven proton transport were assayed in tonoplast fractions isolated from the roots (Figure 10). It was shown that SA does not induce significant changes in both V-ATPase activities, suggesting that this hormone is not involved in V-ATPase regulation.

**Figure 11:**
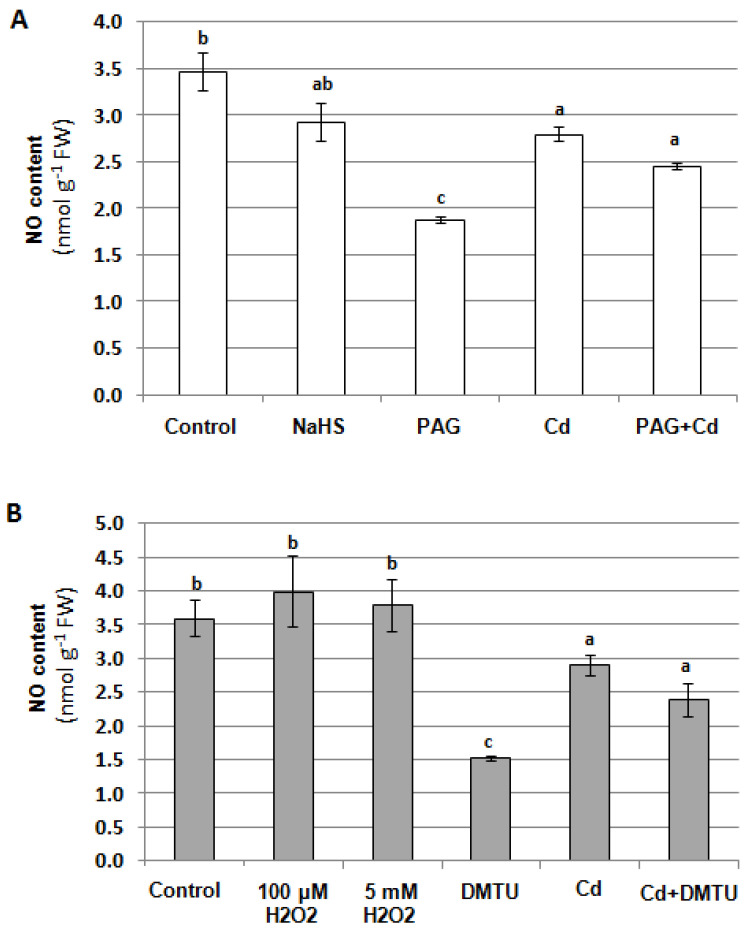
Effects of H_2_S (**A**) and H_2_O_2_ (**B**) on NO content in cucumber roots. Plants were treated with 100 μM CdCl_2_, 100 μM NaHS (H_2_S donor), 1 mM propargylglycine (PAG, inhibitor of H_2_S biosynthesis), 100 μM H_2_O_2_, 5 mM H_2_O_2_, and 5 mM dimethylthiourea (DMTU, H_2_O_2_ scavenger), or were grown without these compounds (control) for 24 h. The results presented are the averages of 3–6 replicates ± SE. Different letters represent homogeneous groups according to Tukey’s test (*p* < 0.05).

In our previous studies, H_2_O_2_ and H_2_S were shown to affect V-ATPase activity [7]. The possible interactions between NO and H_2_O_2_ as well as H_2_S were assayed in the roots of unstressed and Cd-treated cucumber plants. To determine the contribution of both molecules to NO generation, seedlings were exposed to 100 μM NaHS (H_2_S donor), 1 mM propargylglycine (PAG, H_2_S biosynthesis inhibitor), 100 μM and 5 mM H_2_O_2_, and 5 mM dimethylthiourea (DMTU, H_2_O_2_ scavenger) for 24 h. It was found that the reduction in the endogenous H_2_S level using PAG caused a decrease in the NO content (by about 50%) only in the roots of plants growing under normal conditions (Figure 11A). Similarly, the NO level was lowered by 60% in the control plants after their treatment with DMTU. However, under cadmium stress, changes in H_2_O_2_ content did not affect endogenous NO level (Figure 11B). Therefore, it seems that the Cd-induced reduction in NO level does not depend on H_2_S or H_2_O_2_ levels.

**Figure 12:**
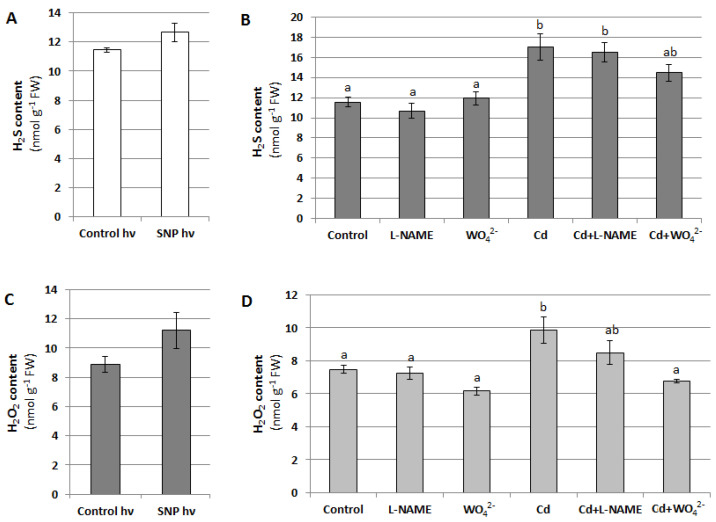
Effect of NO on H_2_S (**A**,**B**) and H_2_O_2_ (**C**,**D**) levels in cucumber roots. Plants were treated with SNP, Cd or inhibitors as described in Figure 5. The results presented are the averages of 4–6 replicates ± SE. (**A**,**C**) There were no statistically significant differences between the controls and SNP treatment (independent-sample *t*-test, *p* < 0.05). (**B**,**D**) Different letters represent homogeneous groups according to Tukey’s test (*p* < 0.05).

On the other hand, during signal transduction, NO can act upstream of H_2_S and H_2_O_2_, regulating their concentration in the cell. For this reason, H_2_S and H_2_O_2_ content was measured in cucumber seedlings treated with SNP, L-NAME, WO_4_^2^**^−^**, and Cd for 24 h. It was shown that changes in the NO level, generated using NO donor or two inhibitors, had no effect on H_2_S concentration in the roots of plants unstressed and stressed with Cd (Figure 12A,B). Under the same conditions, the H_2_O_2_ content also did not change significantly. The only exception was the reduction in the H_2_O_2_ level observed in plants treated with both WO_4_^2^**^−^** and Cd compared with those exposed to Cd alone (Figure 12C,D). This result suggests that under Cd stress, the decrease in the NO level resulting from NR inhibition may cause a decrease in H_2_O_2_ content.

## 3. Discussion

Flexible adaptation to changing environment requires efficient extracellular and intracellular signal transduction cascades mediated by secondary messengers such as Ca^2+^, phytohormones, or NO. The role of NO as a signaling molecule has been confirmed in many plant species. It participates in both developmental and growth processes (e.g., [35,36,37,38], as well as in reactions to biotic [39,40] and abiotic [41,42,43,44] stresses, including Cd stress [20,21,22,23]. Interestingly, an excellent review by Meng et al. [45] clearly showed that there is no universal pattern of changes in NO content in plant tissues under Cd stress conditions. The NO level may increase or decrease in different plant organs/cell cultures depending on the species, Cd concentration, and duration of stress. Moreover, endogenous NO has been found to negatively and positively affect plant adaptation to Cd stress, although in most cases, this molecule enhances Cd tolerance [45]. Therefore, it is important to clarify the role of NO in various stages of plant response to the presence of Cd, especially to determine which signaling molecules interact with NO in this process and which factors are responsible for the positive or negative effect of NO donors on plant metabolism.

Our results suggest that, as in many other plant species [45], NO plays an essential function in Cd stress perception and/or acquiring stress tolerance in *C. sativus*, since the level of this molecule decreases in *C. sativus* roots after 24-h CdCl_2_ application. This effect is dose-dependent and is observed only at higher metal concentrations (Figure 1 and Figure 2). Under such conditions, a reduction in seedling elongation was observed (Figure 3 and Figure S1), whereas longer exposure to Cd caused visible symptoms of impaired growth and development (Figure S2). Similarly, in barley roots, it has been shown that a lower concentration of Cd (10–20 μM) stimulates NO production, whereas a higher level, in the range of 50–60 μM, decreases it [46].

Nitrate reductase has been proposed as one of the main NO sources in plant cells [29]. Moreover, it was confirmed that increased NO generation, observed in the roots of cucumber seedlings treated with salt stress, was related to NR activity [47]. Thus, one of the reasons for the reduction in NO content may be Cd-dependent downregulation of nitrate reductase activity (Figure 4). This is a common phenomenon [48,49,50,51] and it seems to be a universal plant response to this heavy metal, since there are no literature data demonstrating other Cd effects on NR activity, i.e., an increase or no change. In this study, actual NR activity, modulated by phosphorylation and binding of the 14-3-3 protein [52], was more sensitive to Cd exposure and its downregulation was observed even at a low Cd concentration (10 µM). Higher concentrations of this metal also affected total NR activity (Figure 4). This effect may be the result of a direct Cd interaction with the enzyme substrate-binding site, as proposed by Singh et al. [51]. Alternatively, Cd can interfere with electron transport via the NADH-heme pathway or decrease the total NR protein level [52].

On the other hand, the observed decrease in the NO level was not strictly related to the reduction of NR activity. Changes in enzyme activity were more pronounced than the modulation of endogenous NO. It is well known that NO production is a side effect of NR action, estimated at approximately 1% of the basic enzyme activity [27]. Moreover, it is possible that Cd disturbs other NO-generating pathways, including NOS-like activity, [53] or affects NO scavenger systems, such as hemoglobins, especially class I nonsymbiotic hemoglobins [54] or S-nitrosothiols, particularly nitrosoglutathione (GSNO), the main donor and reservoir of NO [55]. NOS-like activity was found to be inhibited by Cd in wheat roots [53], whereas NO and GSNO levels, as well as GSNOR reductase activity, decreased in pea leaves under Cd stress conditions [56].

One of the negative effects of Cd on plant cells is the downregulation of vacuolar H^+^-ATPase [5,6,7], the tonoplast proton pump essential for proper plant growth [57]. However, the mechanism of action of Cd on this enzyme has not yet been fully elucidated. The present study showed that inhibitors of both NR and NOS-like enzymes (tungstate and L-NAME, respectively) decreased V-ATPase activity under control conditions as well as under Cd stress. In contrast, the application of a NO donor (SNP) had the opposite effect; stimulation of V-ATPase was observed (Figure 5). Thus, NO is suggested to be a positive V-ATPase regulator in cucumber roots and different NO synthesis pathways may be involved in this regulation. These results are consistent with those reported by Zhang et al. [58] for V-ATPase in corn leaves. It has been shown that this enzyme is stimulated by 100 μM SNP and the effect is maintained in plants exposed to salt stress [58]. Similarly, in white clover, 100 μM CdCl_2_ inhibited the activity of tonoplast ATPase in the roots, and the heavy metal effect could be partially abolished by adding SNP to the medium or increased by L-NAME application. Interestingly, under the same conditions, the enzyme was regulated in the opposite manner in clover shoots. Its activity increased after plant treatment with Cd, while the addition of SNP together with Cd reduced it in comparison to plants exposed to heavy metal only [10,11].

On the other hand, experiments with animal V-ATPase from mouse macrophages [59] or synaptic vesicles of rat brains [60] revealed negative regulation of the enzyme by NO donors. It should be noted, however, that in both studies, NO donors were introduced into the medium containing macrophages or synaptic vesicles for a short time period, from 20 to 40 min, before measuring V-ATPase activity. Therefore, NO released acted directly and at higher concentrations on the enzyme present in the membranes [59,60]. In plant research, the SNP effect was long-lasting, ranging from 24 h in *Cucumis* (Figure 5) to 14 d in *Trifolium* [11]. Additionally, SNP was introduced into the plant growing medium and NO had an indirect influence on enzyme activity.

To explain the mode of NO action on cucumber V-ATPase, the expression of selected *VHA* genes as well as the protein level of the three enzyme subunits were analysed. However, after SNP application, some changes such as a decrease in *VHA-A* and *VHA-B* mRNA level were observed (Figure 6A), which were not coupled with a decrease in the protein level of VHA-A and VHA-B (Figure 7A,B). Another possibility, not verified in this study, is the NO-dependent post-translational modification of V-ATPase, such as S-nitrosylation or Tyr nitration. S-nitrosylation of the VHA-A subunit has been detected in *Arabidopsis*, but the role of this modification in the regulation of pump activity has not been clarified [61,62]. In animal cells, Swallow et al. [59] suggested that S-nitrosylation inhibits enzyme activity. It seems likely that the responses of plant and animal vacuolar proton pumps to NO may vary due to a slightly different protein structure, including a distinct profile of Cys residues in VHA subunits, as well as different enzyme locations and physiological roles [63].

NO-induced modulations of plant V-ATPase activity are probably not the result of a direct action of this molecule on the enzyme, but rather the general effect of NO on plant metabolic pathways and, consequently, on pump activity. The opposite effect of SNP on V-ATPase in clover roots, directly exposed to the NO donor, and in the aboveground part of the plant, with no contact with this compound, seems to confirm this assumption [10,11]. Moreover, it was suggested that SNP action is based on its ability to restore the correct endogenous NO level, rather than increasing the NO content in plant tissues, as NO released from SNP regulates its own cellular homeostasis. It was shown that S-nitrosylation inhibits the activity of nitrate reductase [64], nitrite reductase [65], and GSNO reductase [66].

In *C. sativus* roots, the Cd-induced decrease in NO level occurs rapidly within 2 h of stress initiation (Figure S3). This change can be perceived as a signal. Since V-ATPase activity was measured after 24 h of Cd treatment, it is possible that in signaling pathways regulating pump activity, NO can act upstream or downstream of other intercellular messengers, including phytohormones. Many studies have indicated that exposure of plants to Cd leads to modulation of endogenous level of various phytohormones [67,68]. A decrease in the content of some of them is a symptom of metal toxicity, whereas an increase in the level of others, such as ethylene, jasmonic acid, salicylic acid, and to a lesser extent ABA and brassinosteroids, is an element of plant adaptation to heavy metal stress [69]. Our results indicated that SA may be a hormone involved in the response of cucumber root cells to Cd (Figure 8C). In addition, the increase in SA content was found to be biphasic and correlated with enhanced level of its precursor, BeA, in the second phase, between 8 and 24 h of the stress reaction (Table 1, Figure S4). This suggests that the initial increase in SA level may be related to the release of this hormone from inactive conjugates, followed by enhanced SA biosynthesis.

Relatively little information is available in the literature regarding changes in endogenous SA levels in plants grown under Cd stress conditions; however, an increase in SA content in response to Cd has been confirmed in *Noccae apraecox* [70], *Arabidopsis* [71], and wheat [72]. In pea leaves, 50 μM CdCl_2_ decreased SA levels, but strongly increased methyl salicylate content [73]. In *Kosteletzkya virginica*, reduced or increased SA accumulation was observed, depending on the duration of heavy metal treatment [74]. Tao et al. [71] showed that elevated endogenous SA levels intensify Cd stress in *Arabidopsis*. On the other hand, numerous studies have demonstrated that exogenous SA can induce Cd tolerance. This was associated with increased activity of antioxidant enzymes and reduced accumulation of H_2_O_2_ [75,76,77,78], stimulation of L-cysteine desulfhydrase and elevation in H_2_S content [79], restoration of hormone homeostasis, including modulation of ABA, IAA, and cytokinin levels, disturbed by Cd, dehydrin accumulation, activation of phenylalanine ammonia-lyase (PAL), and stimulation of lignin biosynthesis to reduce Cd penetration inside the cell [80], as well as limiting Cd uptake and translocation to the aboveground parts of the plant [78].

It is well known that V-ATPase is involved in plant stress responses. This is related to its ability to generate an electrochemical H^+^ gradient across the vacuolar membrane, which drives secondary transporters responsible for the sequestration of mineral compounds, osmoprotectants, and toxic ions such as Na^+^ and Cd^2+^ inside the vacuole [81]. Under some unfavorable conditions, including salt stress, SA application upregulates V-ATPase in the roots and leaves of *Trachyspermum ammi* [82]. Thus, we hypothesized that SA could also modulate V-ATPase activity in cucumber. Unfortunately, no changes in V-ATPase activity were observed in the roots of seedlings treated with SA (Figure 10), although SA increased the content of NO (Figure 9), which acts as a positive regulator of the pump (Figure 5). It is possible that exogenous SA induces NO generation independently of nitrate reductase and NOS-like activity, which have been shown to be involved in the positive regulation of the enzyme (Figure 5C). However, literature data indicate that in other plant species, SA can stimulate NR activity [83] as well as NOS-like activity [84].

Another possibility for the interaction between SA and NO is the modulation of hormone level by this signaling molecule. Some data confirm that SA may act downstream of NO. In tobacco leaves, NO increases SA synthesis by stimulating *PAL* gene expression via a cGMP- and cADPR-dependent pathway [85]. It was also shown that treatment of *Arabidopsis* with NO increases SA content in leaves [86], while *Arabidopsis* lines with silencing of the hemoglobin encoding gene are characterized by increased levels of both NO and SA in their tissues [87]. In this study, no relationship between NO and SA was observed in plants grown under control conditions. However, under Cd stress, interrelations between NR activity and SA level, as well as between NOS-like activity and BeA content, were demonstrated. Our results suggest that NO generated by NR increases the concentration of SA in cucumber roots, whereas NO produced from L-Arg inhibits SA biosynthesis at the stage preceding BeA formation, possibly affecting SA production (Table 1).

Our earlier research confirmed the interplay between H_2_O_2_ and H_2_S in the roots of cucumber seedlings treated with Cd as well as the role of both molecules in V-ATPase regulation [7]. Since NO has been shown to be another regulator of V-ATPase, the relationship between NO and H_2_S, as well as H_2_O_2_, was verified to expand the previously described signaling pathways leading to the regulation of enzyme activity. The available data indicate that H_2_S acts upstream or downstream of NO in different physiological and developmental processes as well as in response to abiotic stress. Moreover, the functional parallelism between NO and H_2_S signaling has been demonstrated in plants exposed to heavy metals [88]. Similarly, it was found that H_2_O_2_ production occurs in parallel with NO generation in many plant tissues under abiotic and biotic stresses, and both signals can act synergistically or independently [89]. However, the present results showed that H_2_O_2_ and H_2_S had no effect on NO level in cucumber roots under Cd stress, although the reduction in endogenous level of both molecules caused a decrease in NO content in plants grown under control conditions (Figure 11). Additionally, NO did not seem to be involved in the regulation of H_2_S content in cucumber roots. In contrast, NR-related inhibition of NO generation resulted in a decrease in the H_2_O_2_ concentration in plants treated with cadmium (Figure 12). Therefore, it can be suggested that different crosstalk in the NO signaling network occurs in cucumber roots under various conditions.

## 4. Materials and Methods

### 4.1. Plant Growing Conditions

The cucumber (*Cucumis sativus* L. cv. Wisconsin) seeds were germinated on filter paper soaked in distilled water at 25 °C for 2 days in the dark. After germination, seedlings were transferred to a hydroponic culture and cultivated in a nutrient solution consisting of 1.7 mM Ca(NO_3_)_2_, 1.7 mM KNO_3_, 0.33 mM MgSO_4_, 0.33 mM KH_2_PO_4_, 25 μM ferric citrate, 3.3 μM MnSO_4_, 1.7 μM H_3_BO_3_, 0.3 μM CuSO_4_, 17 nM Na_2_MoO_4_, and 3 nM ZnSO_4_ (pH 6.2). After 5 days of growth, the medium was changed to a fresh solution (pH 5.5, control) or solution (pH 5.5) enriched with additional compounds: 10 μM CdCl_2_, 50 μM CdCl_2_, 100 μM CdCl_2_, 150 μM CdCl_2_, 10 µM salicylic acid (SA), 50 µM SA, 10 μM sodium nitroprusside (SNP, NO donor) [90], 50 μM Na_2_WO_4_ (WO_4_^2^**^−^**, nitrate reductase inhibitor) [91], 100 µM N-nitro-L-arginine methyl ester (L-NAME, inhibitor of NOS-like activity) [92], 100 µM H_2_O_2_, 5 mM H_2_O_2_, 5 mM dimethylthiourea (DMTU, H_2_O_2_ scavenger) [93,94], 100 μM NaHS (H_2_S donor), or 1 mM propargylglycine (PAG, inhibitor of H_2_S biosynthesis) [95,96]. The plant growth continued for the next 24 h. In the pretreatment experiments, the medium was changed twice, on the fourth and fifth days of cultivation. The roots of cucumber were collected after 6 days of growth. For long-term observation of the effect of Cd on the cucumber phenotype, after 5 d of growth, some plants were transferred to Cd-containing medium for a period longer than 24 h, up to 19 d. The medium was changed at least once a week. The growing conditions were as follows: 180 μmol photons m^−2^s^−1^ of light, 16/8 h light (25 °C)/dark (22 °C) regime and constant 70% relative humidity.

Plants treated with SNP, which releases NO as a result of photolysis [97], as well as corresponding control plants were placed in glass containers and transferred to continuous light and constant temperature (23 °C) conditions.

### 4.2. Determination of NO, H_2_S and H_2_O_2_ Content

The NO content was measured using both colorimetric and fluorescence methods.

The first method, used according to Bryan and Grisham [98] and Filippou et al. [99] with some modifications, based on the conversion of NO to NO_2_^−^ at low pH and determination of NO_2_^−^ concentration using Griess reagent. Roots (1 g) were ground in a chilled mortar with the addition of 2 mL of cold 4% Zn(CH_3_COO)_2_ dissolved in 50 mM CH_3_COOH-CH_3_COONa buffer (pH 3.6). The homogenate was centrifuged at 12,000× *g* for 20 min at 4 °C. The supernatant was used for the colorimetric reaction. The reaction mixture consisted of 0.5 mL of supernatant, 0.25 mL of 1% sulfanilamide dissolved in 1 N HCl, and 0.25 mL of 0.01% *N*-(1-Naphthyl)ethylenediamine dihydrochloride. After 30 min of incubation, absorbance was measured at 540 nm. The amount of NO was calculated based on the standard curve for nitrite and expressed as nmol g^−1^ fresh weight of the roots.

In the second method, described by Reda et al. [47], NO was detected by imaging the fluorescence related to the transformation of DAF-2D (5,6-diaminofluorescein diacetate) to a triazole derivative of fluorescein. Excised cucumber roots were incubated in the dark in 20 mM HEPES-KOH (pH 7.4) with 10 μM DAF-2D for 10 min at room temperature. NO-related fluorescence was observed using a fluorescence microscope Zeiss Axio Image M2 (Carl Zeiss, Oberkochen, Germany) and a Tag-YFP filter with an emission wavelength of 524 nm.

The H_2_O_2_ and H_2_S level was determined in cucumber roots using the colorimetric methods of Velikova et al. [100] and Li [101], respectively, with some modifications. Both procedures have been described in detail by Kabała et al. [7].

### 4.3. Determination of Phytohormone Content

The contents of plant hormones, including indolyl-3-acetic acid (IAA), *cis*-zeatin (cZEA), *trans*-zeatin (tZEA), gibberellin A1 (GA1), gibberellin A4 (GA4), *cis*, *trans*-abscisic acid (ABA), jasmonic acid (JA), methyl jasmonate (MeJA), SA, and its precursor benzoic acid (BeA), were determined according to Dziurka et al. [102]. Cucumber root extracts enriched with internal isotopic standards (Olchemim, Olomouc, Czech Republic) were analyzed using a UHPLC system (Infinity 1260, Agilent, Santa Clara, CA, USA) with an Ascentis Express RP-Amide analytical column (2.7 μm, 2.1 mm × 75 mm; Supelco by Sigma Aldrich, St. Louis, MO, USA) coupled with a triple quadrupole mass spectrometer (6410 Triple Quad LC/MS, Agilent, Santa Clara, CA, USA) equipped with electrospray ionization. The concentration of phytohormones was presented as ng g^−1^ fresh weight of the roots.

### 4.4. Determination of Protein Level

The protein level was determined according to Bradford method [103]. BSA was used to prepare a standard curve.

### 4.5. Isolation of Tonoplast Fractions and Determination of V-ATPase Activities

Tonoplast vesicles were isolated from cucumber roots according to the procedure described by Kabała and Kłobus [104], using a discontinuous sucrose density gradient (20/28/32/42% (*w*/*w*) sucrose).

The V-ATPase (EC 3.6.3.14) hydrolytic activity was measured in tonoplast vesicles according to the method of Gallagher and Leonard [105] as modified by Kabała et al. [6]. The amount of inorganic phosphate released during the reaction was determined according to Ames [106]. V-ATPase activity was expressed as μg Pi h^−1^ mg^−1^ protein.

ATP-driven proton translocation across the tonoplast was measured spectrophotometrically by monitoring changes in acridine orange absorbance at 495 nm, as described by Kabała et al. [6], and was expressed as ΔA_495_ min^−1^ mg^−1^ protein.

V-ATPase activity showed high variability in control samples; therefore, to compare the data obtained in individual analyses, the results were presented as relative values (% of control).

### 4.6. Determination of Nitrate Reductase Activity

Nitrate reductase (EC 1.7.1.1) activity was determined based on the method described by Reda et al. [107] with some modifications. Roots (1.5 g) were homogenized in a chilled mortar with 1 mM PVPP and 2 mL of cold 50 mM HEPES-KOH buffer (pH 7.5) containing 1 mM DTT, 1 mM PMSF, and 1% BSA. The homogenate was centrifuged at 15,000× *g* for 15 min at 4 °C. The supernatant was used for the colorimetric reaction.

Two types of NR activities were determined: total activity (measured in the presence of EDTA) and actual activity (measured in the presence of MgCl_2_). The reaction mixture consisted of 350 μL of 143 mM HEPES-KOH buffer (pH 7.5), 50 μL of 200 mM KNO_3_, 50 μL of 100 mM MgCl_2_ or 50 μL of 100 mM Na_2_EDTA, 250 μL of water, and 200 μL of the supernatant. The reaction was started by adding 33 µL of 5.8 mM NADH, run at 27 °C for 15 min and terminated with 67 µL of 1 mM zinc acetate. The samples were centrifuged for 10 min at 10,000× *g*. The NO_2_^−^ level in the resulting supernatant was determined using sulfanilamide and *N*-(1-naphthyl)ethylenediamine dihydrochloride as described above. Enzyme activity was expressed as µmol of NO_2_^−^ h^−1^ g^−1^ fresh weight.

### 4.7. Total RNA Extraction, cDNA Synthesis and Gene Expression Analysis

Total RNA was isolated using EXTRAzol (BLIRT, Gdańsk, Poland), according to the manufacturer’s instructions. After RNase-free DNase I (Thermo Fischer Scientific, Waltham, MA, USA) digestion, RNA samples were used to cDNA synthesis (High-Capacity cDNA Reverse Transcription Kit, Applied Biosystems, Foster City, CA, USA). The expression of selected *VHA* genes, encoding V-ATPase subunits, was analyzed using real-time PCR. Reactions were performed in a LightCycler 480 system (Roche, Basel, Switzerland) with a Real-Time 2 × PCR Master Mix SYBR kit (A&A Biotechnology, Gdańsk, Poland). Tonoplast intrinsic protein 41-like (*TIP41*) and elongation factor 1-alpha (*EF1*) were chosen as reference genes, according to Migocka and Papierniak [108].

The qPCR reaction conditions were as follow: 30 s at 95 °C; 40 cycles of: 10 s at 95 °C, 10 s at 58 °C (for *CsVHA-A*, *B*, *c2*, *c3*, *a1*, *a3*), 60 °C (for *CsVHA-c1*) or 66 °C (for *CsVHA-a2*), and 12 s at 72 °C; 15 s at 65 °C (for *CsVHA-A*, *B*, *a1*, *a3*, *c2*, *c3*), 68 °C (for *CsVHA-c1*) or 72 °C (for *CsVHA-a2*), and 30 s at 40 °C of final cooling. LightCycler software 4.1 (Roche, Basel, Switzerland) was used for data analysis. The sequences of the primers used in the reaction were consistent with those used in our previous publication [7].

### 4.8. Immunoblotting and Coomassie Staining

Cucumber roots were powdered in liquid nitrogen. Frozen tissue (500 mg) was mixed with 400 μL of extraction buffer consisting of 50 mM Tris-HCl (pH 7.5), 150 mM NaCl, 1 mM EDTA, 1 mM phenylmethylsulfonyl fluoride and 1× protease inhibitor cocktail (cOmplete, Roche, Basel, Switzerland), according to the modified method of Jurado–Flores et al. [109]. The samples were centrifuged for 5 min at 5000 rpm at 4 °C to collect the supernatant and were centrifuged again. This step was repeated several times to obtain clear protein fractions.

Total root proteins (10 µg) were mixed with the Laemmli buffer. After denaturation, the samples were separated by electrophoresis. Then, 4% stacking and 10% separating polyacrylamide gels were used. The BioBLU Prestained Protein Ladder (Bio-Rad, Hercules, CA, USA) was used as a molecular weight marker. After separation, each gel was divided into two parts, one without proteins of interest, which was used for staining with Coomassie Brilliant Blue R-250, and the other for protein transfer to the membrane and Western blot analysis.

After electrotransfer and membrane blocking with 5% milk dissolved in phosphate-buffered saline with the addition of 0.1% (*v*/*v*) Tween 20 (1 h), samples were incubated overnight at 4 °C with polyclonal antibodies (Agrisera, Vännäs, Sweden) against the subunits A (AS09 467), B (AS14 2775), or E (AS07 213) of V-ATPase, diluted 1:5000 in milk solution. The next day, the samples were treated with secondary antibodies conjugated with horseradish peroxidase (Goat Anti-Rabbit IgG (H+L)-HRP Conjugate, Bio-Rad, Hercules, CA, USA). The secondary antibodies were diluted to 1:10,000 and applied for 1 h. The Amersham ECL Select Western Blotting Detection Reagent (Cytiva, Marlborough, CT, USA) and ChemiDoc Imaging Systems (Bio-Rad, Hercules, CA, USA) were used for blot and gel analyses.

### 4.9. Statistical Analysis

Statistical analyses were performed with Statistica (TIBCO Software Inc., Palo Alto, CA, USA), version 13.3. The results were considered statistically significant at *p* < 0.05. The normality of the data was verified using the Shapiro–Wilk test. Statistically significant differences in V-ATPase activity, presented as % of the control, were evaluated with a one-sample *t*-test. The homoscedasticity of the data was checked using the Brown–Forsythe test. The comparison of two groups of data was performed using the independent-sample *t*-test, while one-way ANOVA with Tukey’s test for post hoc analysis was used to compare more than two groups of data. At least three biological replicates were performed in all experiments and the results are presented as mean values ± standard error (SE). Detailed information regarding the tests used and the number of replicates is available in the figure legends.

## 5. Conclusions

In this study, it was found that both exogenously applied NO (in the form of SNP) and endogenous NO stimulated V-ATPase activity in cucumber roots under control and Cd stress conditions. Both NO synthesized from L-Arg via a NOS-like pathway and NO produced from NO_3_**^−^** by NR may be involved in this regulation. The mechanism of NO action on V-ATPase is not related to changes in the gene expression or protein level of the main V-ATPase subunits. In cucumber roots, Cd induces an increase in SA content, but does not affect the level of JA, MeJA, ABA, IAA, cZEA, tZEA, GA1, and GA4. Under control and Cd stress conditions, NO signaling appears to interact with the SA pathway and a positive correlation between these molecules has been shown. However, the effect of SA on V-ATPase activity has not been confirmed. Only under Cd stress, NR-related NO and its crosstalk with SA and H_2_O_2_ have been suggested to play a key role in the functioning of the signaling network in cucumber roots.

## Figures and Tables

**Table 1:** Effect of NO on salicylic acid (SA) and benzoic acid (BeA) content in cucumber roots. (Plants were treated with SNP, Cd and NO synthesis inhibitors as described in Figure 5. Results presented are averages of five replicates ± SE. Statistically significant differences (independent-sample *t*-test) between the control and SNP or Cd treatments are marked as ^, and between Cd and the corresponding inhibitor treatments as * (*p* < 0.05)).

Treatment	BeA Concentration (ng g^−1^ FW)	SA Concentration (ng g^−1^ FW)
Control (hν)	67.1 ± 2.6	179.4 ± 6.2
SNP (hν)	59.0 ± 3.4	190.7 ± 10.8
Cd	74.0 ± 0.8 ^	252.9 ± 23.1 ^
Cd+L-NAME	83.8 ± 4.1 *	203.3 ± 3.3
Cd+WO_4_^2−^	71.6 ± 4.1	185.5 ± 5.9 *

## Data Availability

The data presented are available in this manuscript and Supplementary Materials.

## Supplementary Materials

The following supporting information can be downloaded at: https://www.mdpi.com/article/10.3390/plants12152884/s1, Figure S1: Images of cucumber seedlings treated with different concentrations of CdCl_2_ for 24 h. Figure S2: Effect of longer treatment of cucumber seedlings with 100 µM CdCl2 on their phenotype. Figure S3: Changes in NO content over time in cucumber roots treated with 100 µM CdCl_2_. Figure S4: Changes in BeA (A) and SA (B) level over time in cucumber roots treated with 100 µM CdCl_2_.
