# Supplementary material for: Involvement of NO in V-ATPase Regulation in Cucumber Roots under Control and Cadmium Stress Conditions
Source: Plants (Basel). 2023 Aug 7;12(15):2884. doi: 10.3390/plants12152884 (PMC10420687; doi:10.3390/plants12152884)
Supplement: Supplementary file 1 [file plants-12-02884-s001.zip › plants-2541549-supplementary.pdf]

Supplementary material

Involvement of NO in V-ATPase regulation in cucumber roots under control and cadmium stress conditions

Magdalena Zboińska [1], Anna Janeczko [2] and Katarzyna Kabała [1,*]

[1] Department of Plant Molecular Physiology, Faculty of Biological Sciences, University of Wrocław, Kanonia 6/8, 50-328 Wrocław, Poland; magdalena.zboinska@uwr.edu.pl (M.Z.); katarzyna.kabala@uwr.edu.pl (K.K.)

[2] The Franciszek Górski Institute of Plant Physiology, Polish Academy of Sciences, Niezapominajek 21, 30-239 Krakow, Poland; a.janeczko@ifr-pan.edu.pl (A.J.)

* Correspondence: katarzyna.kabala@uwr.edu.pl

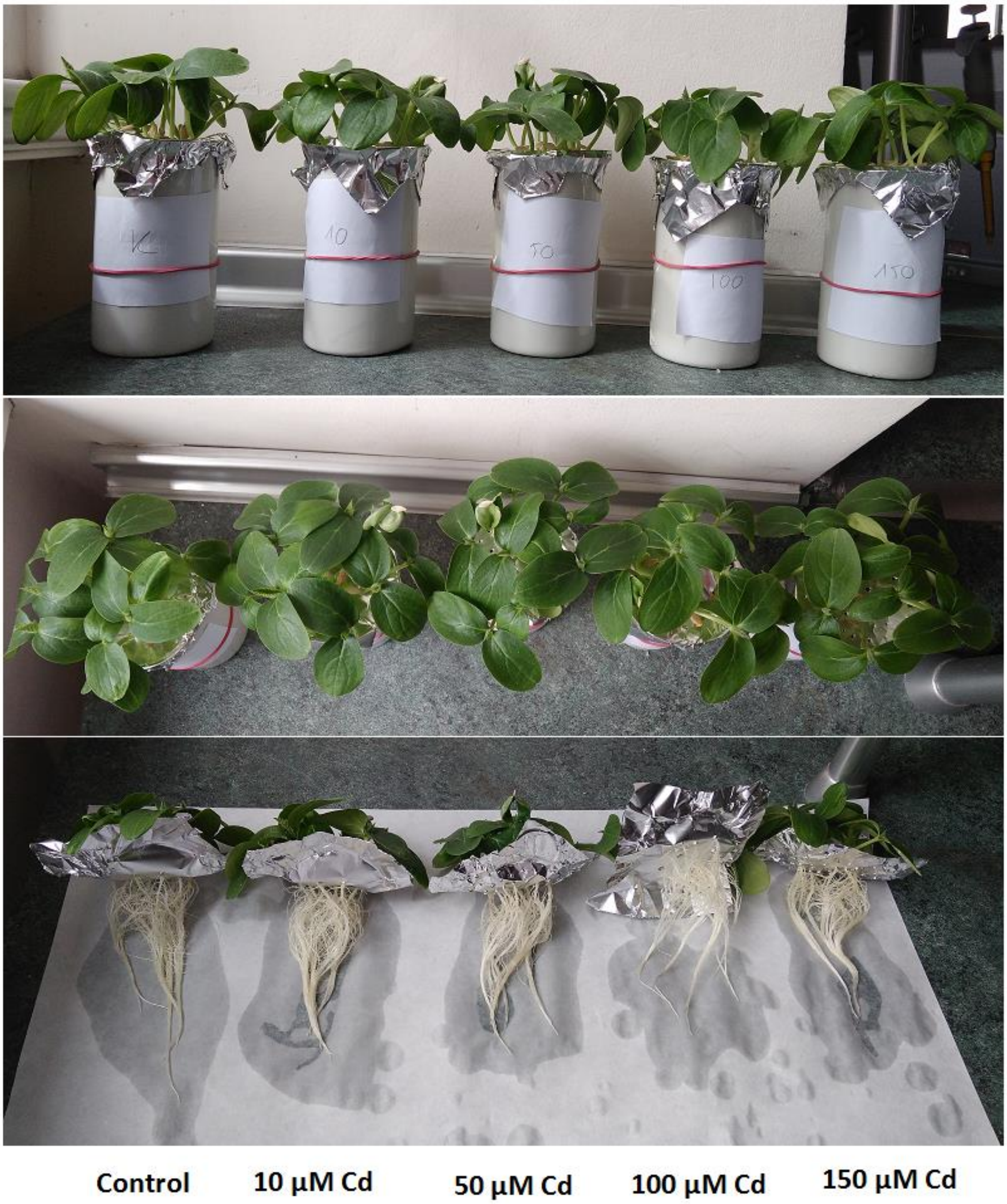

Figure S1. Images of cucumber seedlings treated with different concentrations of $CdCl_2$ for 24 h. No visually significant changes in the phenotype were observed.

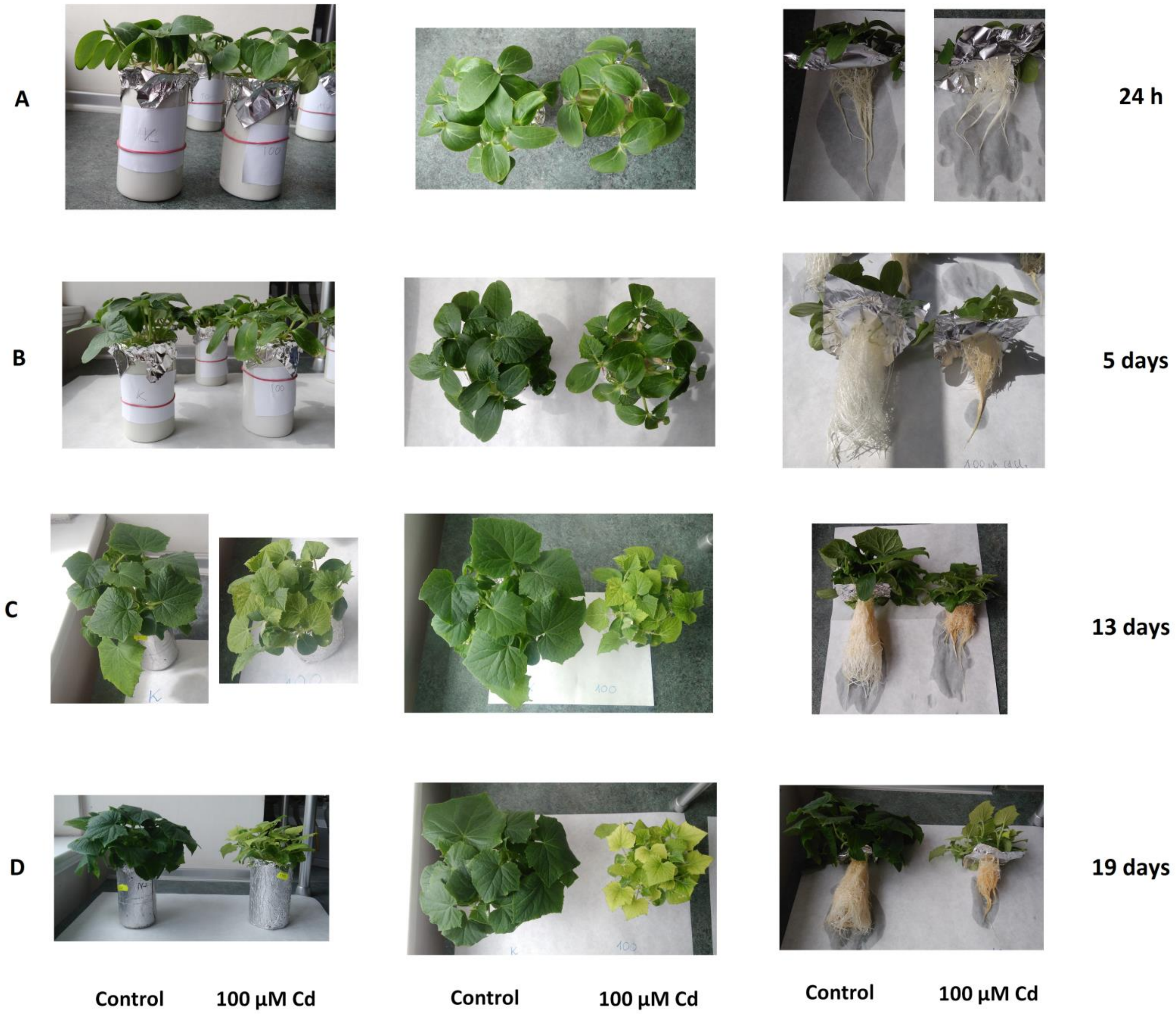

Figure S2. Effect of longer treatment of cucumber seedlings with 100 µM $CdCl_2$ on their phenotype.

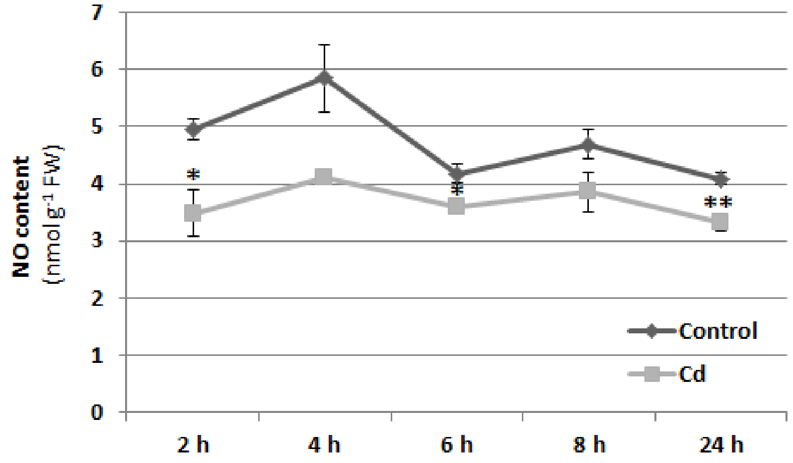

Figure S3. Changes in NO content over time in cucumber roots treated with 100 μM $CdCl_2$. Data represent the means of 3 biological repetitions ± SE. Statistically significant differences (independent-sample t-test) between the control and Cd treatment are marked as * (0.01≤p<0.05) or ** (p<0.01).

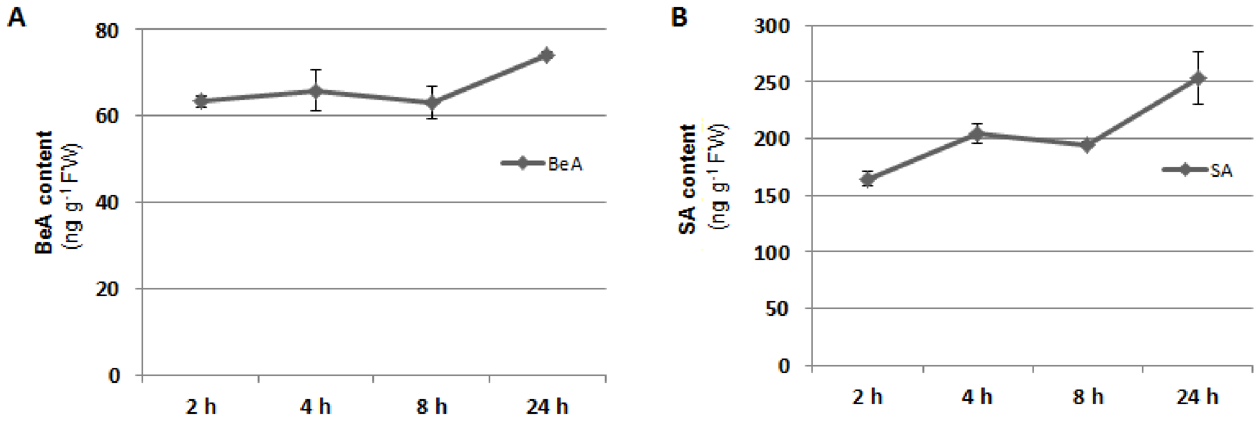

Figure S4. Changes in BeA (A) and SA (B) levels over time in cucumber roots treated with 100 μM $CdCl_2$. Data represent the means of 5 biological repetitions ± SE.
